# Identification of potential specific biomarkers and key signaling pathways between osteogenic and adipogenic differentiation of hBMSCs for osteoporosis therapy

**DOI:** 10.1186/s13018-020-01965-3

**Published:** 2020-09-23

**Authors:** Jianjun Wu, Peian Cai, Zhenhui Lu, Zhi Zhang, Xixi He, Bikang Zhu, Li Zheng, Jinmin Zhao

**Affiliations:** 1 Guangxi Engineering Center in Biomedical Material for Tissue and Organ Regeneration, The First Affiliated Hospital of Guangxi Medical University, Nanning, 530021 China; 2 Guangxi Collaborative Innovation Center for Biomedicine, The First Affiliated Hospital of Guangxi Medical University, Nanning, 530021 China; 3 Department of Orthopaedics Trauma and Hand Surgery, The First Affiliated Hospital of Guangxi Medical University, Nanning, 530021 China; 4 Guangxi Key Laboratory of Regenerative Medicine, The First Affiliated Hospital of Guangxi Medical University, Nanning, 530021 China

**Keywords:** Osteoporosis, Osteogenesis, Adipogenesis, Bone marrow mesenchymal stem cells, Microarray analyses

## Abstract

**Background:**

The differentiation of bone mesenchymal stem cells (BMSCs) into adipogenesis (AD) rather than osteogenesis (OS) is an important pathological feature of osteoporosis. Illuminating the detailed mechanisms of the differentiation of BMSCs into OS and AD would contribute to the interpretation of osteoporosis pathology.

**Methods:**

To identify the regulated mechanism in lineage commitment of the BMSCs into OS and AD in the early stages, the gene expression profiles with temporal series were downloaded to reveal the distinct fates when BMSCs adopt a committed lineage. For both OS and AD lineages, the profiles of days 2–4 were compared with day 0 to screen the differentially expressed genes (DEGs), respectively. Next, the functional enrichment analysis was utilized to find out the biological function, and protein-protein interaction network to predict the central genes. Finally, experiments were performed to verify our finding.

**Results:**

FoxO signaling pathway with central genes like FoxO3, IL6, and CAT is the crucial mechanism of OS, while Rap1 signaling pathway of VEGFA and FGF2 enrichment is more significant for AD. Besides, PI3K-Akt signaling pathway might serve as the latent mechanism about the initiation of differentiation of BMSCs into multiple lineages.

**Conclusion:**

Above hub genes and early-responder signaling pathways control osteogenic and adipogenic fates of BMSCs, which maybe mechanistic models clarifying the changes of bone metabolism in the clinical progress of osteoporosis. The findings provide a crucial reference for the prevention and therapy of osteoporosis.

## Introduction

Osteoporosis, characterized by an increase of bone fragility and susceptibility to fracture owning to low bone mass and degeneration of bone microstructure, is a major social problem in the elderly population [1]. Currently, most therapeutic strategies for osteoporosis were administrated by anti-absorption drugs including bisphosphonates, calcitonin and estrogen [2–5]. Although these drugs have a certain effect on delaying the progress of osteoporosis, the serious side effects caused by a long-term use are concerns of clinical application [6–8]. Thus, it is still an urgent need to discover a safer and more effective therapeutic strategy.

Growing evidences indicated that age-related microenvironmental changes including metabolic changes, immune system diseases, and hormonal disorders, reduced the capacity of osteogenic differentiation and strengthened the ability in adipogenic differentiation of BMSCs, which were the paramount motivators in the initiation and progress of osteoporosis [9–12]. Therefore, a regenerative therapy has aroused much attention, which restores the balance of the normal intraosseous microenvironment of osteoporosis by artificially promoting OS and inhibiting AD from BMSCs. However, the molecular mechanisms of lineage commitment determinants during BMSCs differentiate into OS and AD are still unclear.

Adipogenesis and osteogenesis exist a competitive relation during the differentiation of BMSCs, and the signaling pathways between the adipo-osteogenesis could be altered to favor osteoblast for preventing osteoporosis. Adipo-osteogenic differentiation is a process regulated by multiple factors and signals. It has reported that the activation of glucagon-likepeptide1 (GLP-1) not only promotes osteogenic differentiation of BMSCs, but also inhibits adipogenic differentiation which is beneficial for osteoporosis [13]. Other osteogenic factors such as myocardin-related transcription factor A (MRTFA), γ-glutamyl carboxylase (GGCX), and transducin-like enhancer of split 3 (TLE3) inhibited adipogenic differentiation resulting in amelioration of bone formation [14–16]. Although previous studies have revealed possible molecular mechanisms involved in BMSCs to differentiate into OS and AD, their results are limited to a single lineage and/or a differentiation in late-stages, and the mechanism in the differentiation of BMSCs into OS and AD were still unknown [17–19]. Thus, temporal expression profiles in the early stage of the BMSCs to differentiate into OS and AD may better understand the mechanisms of their lineage commitment determinants.

The rapid development of high-throughput sequencing provides a more systematic and comprehensive approach to studying physiological or pathological mechanisms [20]. Characterizing the features of transcriptional regulation during the different stages of stem cell development would be contributed to clarifying the mechanism of lineage commitment differentiation. Zhang et al. had firstly reported the stage-specific interaction patterns between oocyte and granulosa cells during the development of folliculogenesis with the mRNA-sequencing (mRNA-Seq) technology [21]. Moreover, novel regulators of early cardiomyocyte development from human embryonic stem cells were identified with the temporal transcriptome and methylome [22]. Therefore, bioinformatics analysis is a powerful tool to discover the molecular-level biomarkers and related pathways in the osteogenic and adipogenic differentiation from BMSCs.

In the present study, we utilized the temporal gene expression profiles of an early stage in the in vitro induced (0–4 days) OS and AD from human BMSCs (hBMSCs), combined with bioinformatics and experiments, to explore the potential lineage commitment mechanisms of hBMSCs to differentiate into OS or AD. By comparing the multiple profiles, the hub-genes and key signaling pathways that critical for osteogenic and adipogenic differentiation were identified. Our work might provide the key clues for the mechanism of stepwise osteogenesis and adipogenesis, which would provide a solid reference for the prevention and therapy of osteoporosis.

## Materials and methods

### Affymetrix microarray data source

GSE80614, a gene expression profile of hMSCs differentiated into OS or AD based on GPL6947 platform (Illumina HumanHT-12 V3.0 expression bead chip) were downloaded from the Gene Expression Omnibus (GEO) database. It contains 66 samples including 11 groups in OS or AD differentiation and 3 samples in each group. For the differentiation of BMSCs, the first 2 days are the lineage acquisition phase and the first 2–4 days are the lineage stable phase. So samples in day 0 (0d), day 2 (2d), day 3 (3d), and day 4 (4d) groups of OS or AD were selected to explore the crucial regulators involved in the initial stage of differentiation [23]. More information about the profile could be obtained from the online description (https://www.ncbi.nlm.nih.gov/geo/query/acc.cgi?acc=GSE80614). The study protocols were reviewed and approved by the Ethics Committee of Guangxi Medical University (Nanning, China).

### Screening of differential expression genes (DEGs)

All the data were normalized and analyzed with R-based web application in GEO dataset (GEO2R) [24]. The data was preprocessed with excluding the probes with no gene annotation and selecting the probes with maximum expression value if a gene is corresponding to multiple probes. DEGs were identified by the inclusion criteria with *P* value < 0.05 and fold change (FC) ≥ 2 [25]. The visualization of DEGs was drawn via volcano plot and Venn diagram by using the ggplot2 and VennDiagram packages in R language (version 3.5.1) (http://www.r-project.org/) [26].

### Function enrichment analysis of DEGs

Gene Ontology (GO; http://www.geneontology.org/) analysis, a common and useful method for the computational analysis of large-scale molecular biology and transcriptome data in biomedical research, can classify the genes into three functional categories, including the biological process (BP), cellular component (CC), and molecular function (MF) [27, 28]. Kyoto Encyclopedia of Genes and Genomes (KEGG, http://www.genome.jp/kegg/pathway.html) is a well-knowledge database for understanding the molecular interaction and relation networks [29]. To expound the distinctive biological function, the identified DEGs were utilized to perform the GO term and KEGG pathway enrichment analysis with the database for annotation, visualization, and integrated discovery (DAVID v6.8, https://david.ncifcrf.gov/tools.jsp) online tool [30]. The cutoff value of the result was set at *P* value < 0.05. Scatter plots were used to depict the top 10 and command KEGG pathways in the Image GP (http://www.ehbio.com/ImageGP/), and GO term enrichment analyses were visualized via the bar plot and cord plot with GOplot package in R [31].

### Construction of protein-protein interaction (PPI) networks

Search Tool for the Retrieval of Interacting Genes (STRING, v.10.5; http://string-db.org/cgi/input.pl), an online biological database and web resource, provides integrations and assessments of the PPI which could assess the relationships between DEGs [32]. Score (median confidence) > 0.4 was set as the cutoff criterion of the selected PPIs, followed by PPI network construction via Cytoscape software (version 3.4.0; http://www.cytoscape.org/). The PPI networks, in which each node represents a protein and the lines denote direct interactions between proteins, were then used Between and Stress algorithm of cytoHubba in the Cytoscape to screen the top 20 hub genes in the OS and AD DEGs [33].

### Cell isolation and culture

The hMSCs were isolated from the marrows’ fluid extracted from the patients with traumatic joint replacement based on informed consent. The cells were cultured in Dulbecco’s modified Eagle’s medium (DMEM) containing 10% FBS, penicillin (100 U/ml), and streptomycin (100 mg/ml) underling 37 °C and 5% CO_2_. The medium was replaced every 2 days, and the cells at passage 2 were used for further studies.

### Induction of hBMSCs into OS and AD

Prior to induction, hMSCs with a density of 1.5 × 10^4^/cm^2^ were seeded in growth medium until reach to 90–100% confluence. Then, the medium was changed with the OS or AD induction medium for 4 days. The OS induction medium was prepared with α-MEM that supplemented with 10 mmol/L of glycerol 2-phosphate, 100 nmol/L of dexamethasone, and 50 μmol/L of ascorbic acid. For AD induction medium, 5 μg/ml insulin, 1 μM dexamethasone, 500 μM 3-isobutyl-1-methylxantine and 50 μM indomethacin were added into DMEM [34].

### RNA extraction and quantitative real-time PCR (qRT-PCR)

Intracellular total RNA of the induced samples was purified via a Hipure Total RNA Mini kit (Magen, China) after 0, 2, 3, and 4 days of induction. Next, 1000 ng extracted RNA was reverse transcribed to cDNA using a cDNA synthesis kit (Takara, China). A LightCycler ® 480 Sequence Detention System (Roche, Germany) with PCR Green Master Mix (Roche, Germany) was used to conduct the qRT-PCR for the detection of gene expression. Forkhead box O3 (FoxO3), phosphoinositide-3-kinase regulatory subunit 1(PIK3R1), catalase (CAT), interleukin 6 (IL6), alkaline phosphatase (ALP, an early maker of OS), vascular endothelial growth factor A(VEGFA), fibroblast growth factor 2 (FGF2), and lipoprotein lipase (LPL, an early marker of AD) were normalized by glyceraldehyde-3-phosphate dehydrogenase (GAPDH) as a control. Table 1 lists the primer sequences of the above genes.

**Table 1:** Primer sequences used in qRT-PCR experiments

Primer	Sequence(5′ to3′)
ALPP-F	CCAGATGACTACAGCCAAGG
ALPP-R	GAGTCTCGGTGGATCTCGTAT
PIK3R1-F	TAGTGGTGGACGGCGAAGTA
PIK3R1-R	TTGAGGGAGTCGTTGTGCTG
FoxO3-F	AGTCGGACCCCTTGATGTCT
FoxO3-R	GGTGGTGGAGCAAGTTCTGAT
IL6-F	GTAGTGAGGAACAAGCCAGAGC
IL6-R	GTTGGGTCAGGGGTGGTTAT
CAT-F	AGATAGCCTTCGACCCAAGC
CAT-R	AGCACGGTAGGGACAGTTCA
LPL-F	GGCTGGACGGTAACAGGAAT
LPL-R	CAGCCAGTCCACCACAATGA
VEGFA-F	GAAGGAGGAGGGCAGAATCA
VEGFA-R	GGTCTCGATTGGATGGCAGT
FGF2-F	AGAGCGACCCTCACATCAAG
FGF2-R	AGCCAGGTAACGGTTAGCAC

### Immunofluorescence staining

The expression levels of proteins in the key signaling pathway were detected by immunofluorescence. The cells were removed from the well plate and cleared three times with PBS. After fixed with 4% (w/v) paraformaldehyde for 20 min, the cells were transparented in the Triton X100 for 15 min. Subsequently, endogenous peroxidase activity was blocked by the H_2_O_2_ for 15 min at 25 °C. Then, the cells were incubated with primary antibodies containing PIK3R1 (1:500), FoxO3 (1:100), CAT (1:100), IL6 (1:100), VEGFA (1:200), and FGF2 (1:100) at 4 °C for 12 h according to reagent instructions. After added with secondary antibodies for 1 h at 37 °C, the cells were observed and photographed by using an inverted phase-contrast microscope (OLYMPUS, Japan).

### Statistical analysis

Statistical analysis of the data was performed by one-way ANOVA and the quantitative data were expressed as means ± standard deviation of the experiment. *P* < 0.5 was set as the cutoff of significant differences.

## Results

### DEGs and KEGG pathway of OS induction

**Fig. 1:**
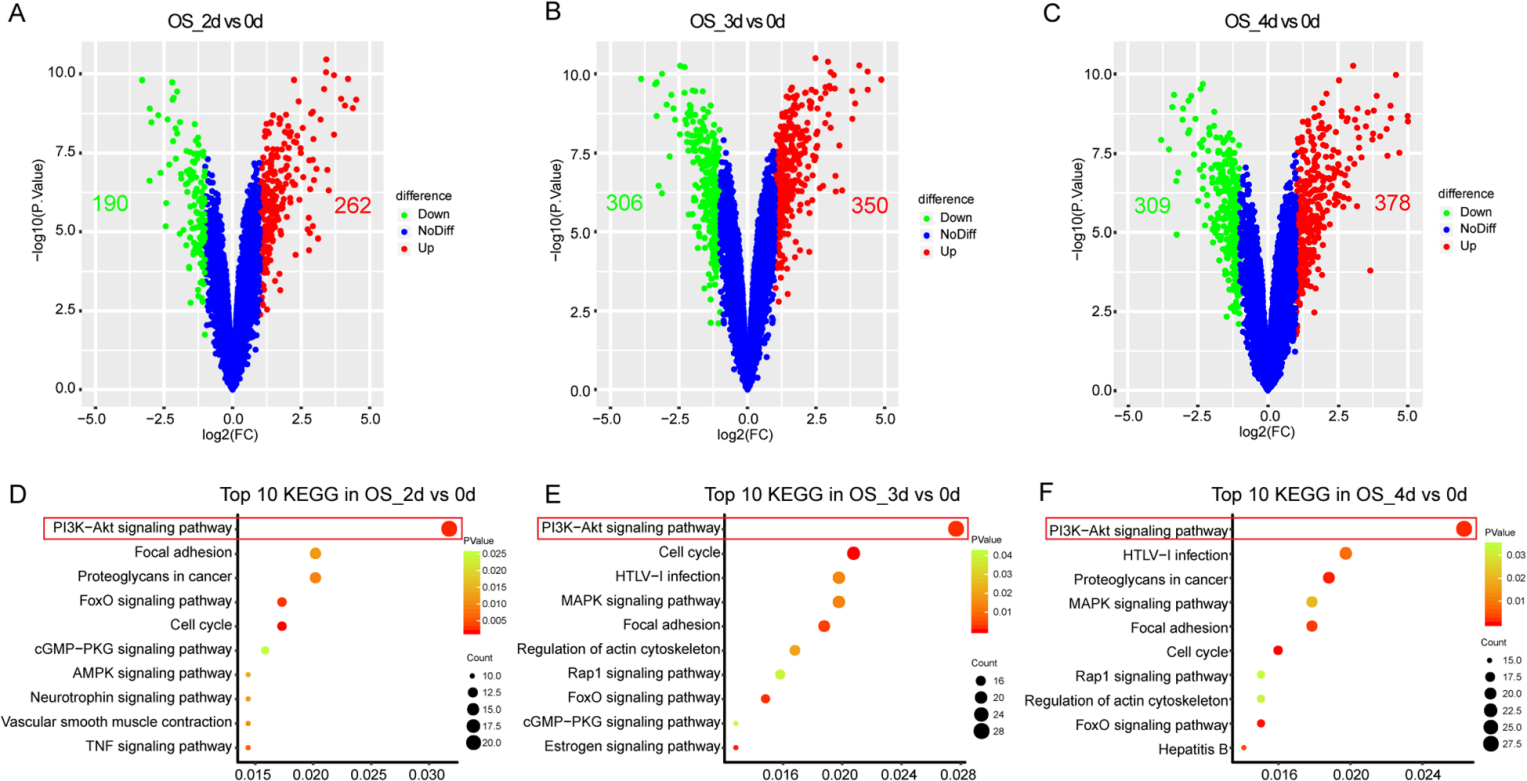
DEGs and KEGG pathway analysis in the three time points of the OS. **a–c** The volcano plot for DEGs of osteogenic differentiation at d2, d3, and d4, respectively. The red point indicated upregulated genes and the green represented downregulation while the blue meant no difference. (DEGs were identified as differentially expressed when P*.*value < 0.05 and fold change (FC) ≥ 2). **d**–**f** Top 10 of DEG enrichment pathway resulted at the at d2, d3, and d4 of the OS, respectively. Point size means the gene numbers while the point color represents the *P* value (*P* value < 0.05). OS osteogenesis, DEGs differentially expressed genes, KEGG Kyoto Encyclopedia of Genes and Genomes, d day

To systematically explore gene expression changes during the early stage of hMSCs differentiated into OS, we identified the DEGs by comparing the OS_2d, OS_3d, OS_4d groups with the group OS_0d. There were 452 DEGs in the OS_2d group, including 262 upregulations and 190 downregulations while 656 DEGs in the OS_3d group, of which 350 were upregulated and 306 were downregulated (Fig. 1a, b). In addition, a total of 687 genes with 378 upregulations and 309 downregulations were detected to be differentially expressed in the OS_4d group (Fig. 1). Moreover, the common upregulations were nearly twice compared to downregulations in these three time points, which might mean that the OS was the result of the upregulation of pro-differentiation genes and activation of related pathways (Figure S1A and B). To explore the potential mechanisms in the OS initiative differentiation, KEGG pathway enrichment analysis was performed with DAVID database. The top 10 pathways sorted by the number of DEGs enriched in every term were displayed via scatter plot (Fig. 1d–f). Obviously, PI3K-Akt signaling pathway was the most significant pathway in each time point during the OS differentiation.

### DEGs and KEGG pathway of AD induction

**Fig. 2:**
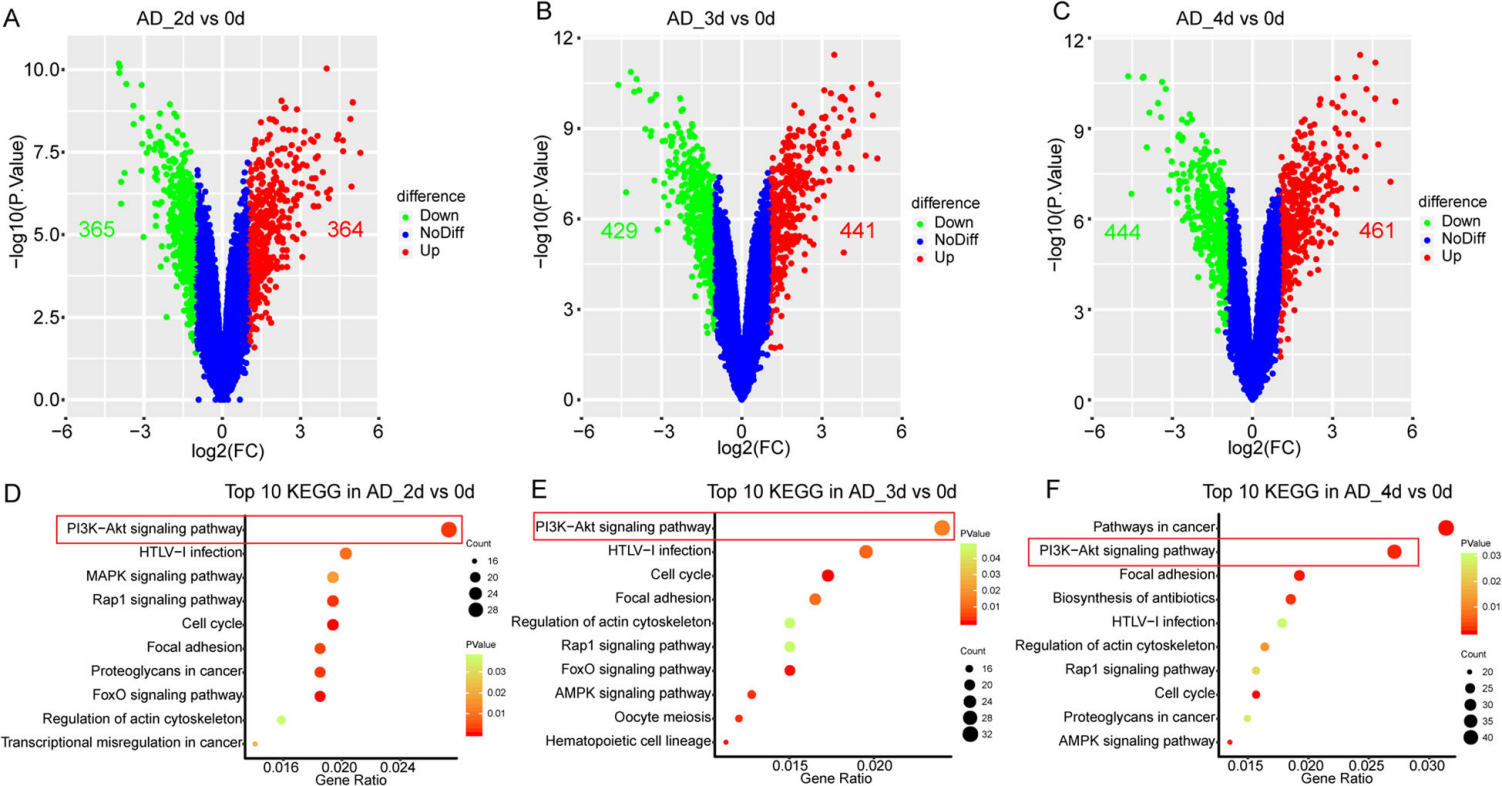
DEGs and KEGG pathway analysis in the three time points of the AD. **a**–**c** The volcano plot for DEGs of adipogenic differentiation at the d2, d2, and d4, respectively. The red point indicates upregulated genes and the green represented downregulation while the blue meant no difference. (DEGs were identified as differentially expressed when *P* value < 0.05 and fold change (FC) ≥ 2). **d**–**f** Top 10 of DEG enrichment pathway resulted at the d2, d3, and d4 of the AD, respectively. The point size meant the gene numbers while the point color represented the *P* value (*P* value < 0.05). AD adipogenesis

There were a total of 729 DEGs in AD_2d group, 870 DEGs in AD_3d group, and 905 DEGs in AD_4d group, including 364, 441, and 461 upregulations while 365, 429, and 444 downregulations in the AD_2d, AD_3d, and AD_4d group, respectively (Fig. 2a–c). The count of DEGs in AD induction showed little change in each time point as well, but increased significantly compared to DEGs OS, which might be due to hMSCs favor differentiate into AD rather than OS [35, 36]. As shown in the Venn diagrams, the number of the downregulations was similar to the upregulations in the AD, but was more than twice that in the OS, meaning the downregulation play more significance in the AD (Figure S1A and B). As the same as OS induction, the top 10 KEGG pathway enrichment at each time point was visualized in the scatter plot (Fig. 2d–f). According to numbers of enriched DEGs, the PI3K-Akt signaling pathway was also ranked at first, ignoring the pathways in cancer usually enriched most genes in the DAVID. Therefore, we surmised that the PI3K-Akt signaling pathway also exerted a crucial effect on AD from BMSCs.

### Key KEGG pathway enrichment

**Fig. 3:**
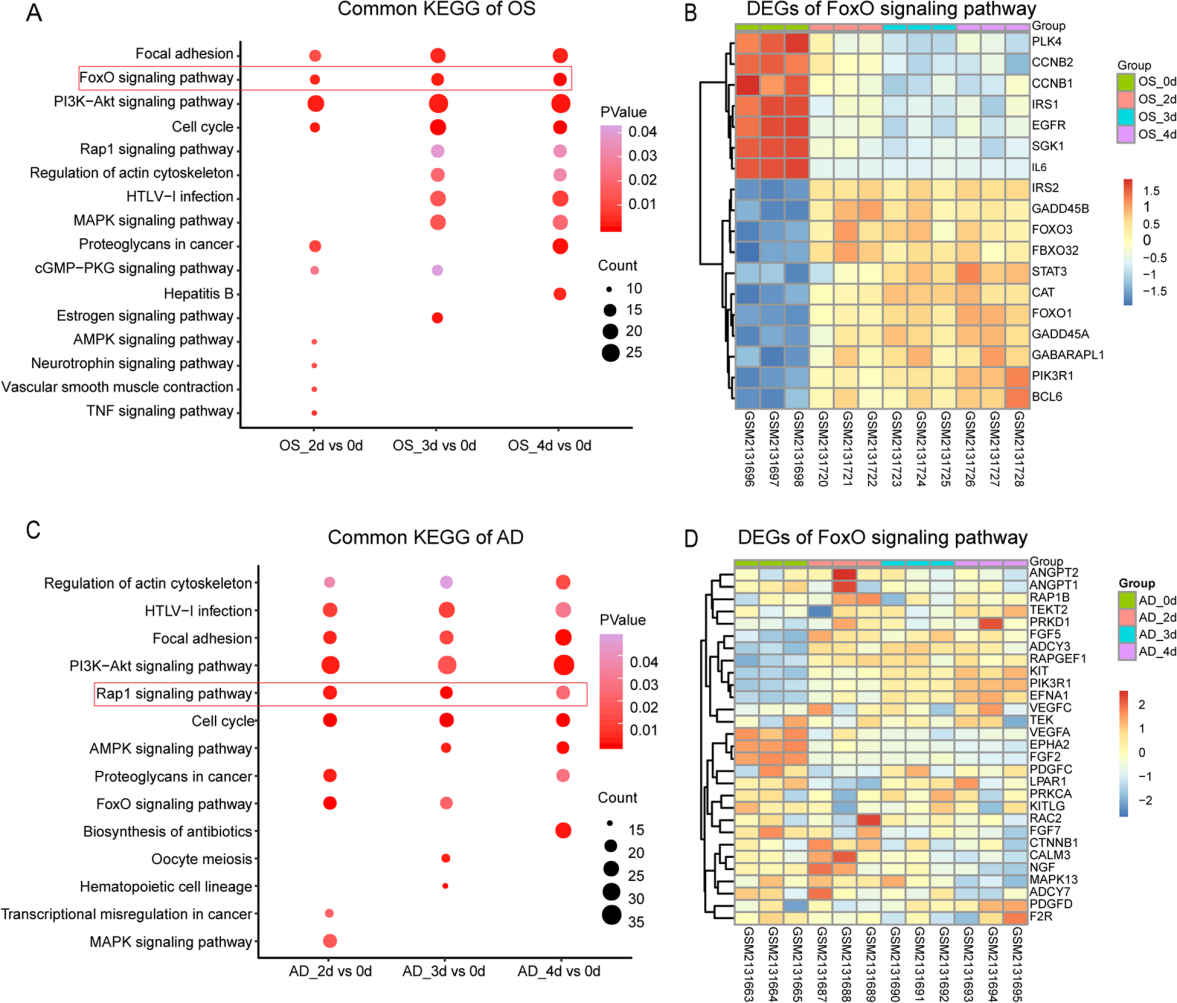
Command pathway analysis. **a** Common enrichment pathway results of the OS in each time point. **b** Hierarchical cluster of the DEGs enriched in the hub pathway (FoxO signaling pathway). The abscissa was the sample, the right-hand indicated gene name, and the left-hand ordinate meant the clustering of DEGs enriched in the key pathway in the OS, while the color represented the log_2_ fold change of expression value (range from bright red (upregulation) to bright blue (downregulation)). **c** Top 10 of each group’s DEG pathway enrichment results of the AD. **d** Hierarchical clustering of the differential genes enriched in the hub pathway (Rap1 signaling pathway). All icons represented the same meaning as above

We next investigated the key signaling pathway in these two lineages of differentiation. The PI3K-Akt signaling pathway, FoxO signaling pathway, focal adhesion, and cell cycle were the common signaling pathways in OS differentiation (Fig. 3a)**.** In AD induction, PI3K-Akt signaling pathway, Rap1 signaling pathway, focal adhesion, regulation of actin cytoskeleton, cell cycle, and HTLV-I infection were all enriched in the three groups (Fig. 3c). Three common pathways including PI3K-Akt signaling pathway, focal adhesion, and cell cycle were all existed in the OS and AD induction. Therefore, we hypothesized that the PI3K-AKT signaling pathway, as well as focal adhesion, and cell cycle were the crucial mechanism for initiating the differentiation of hMSCs. For the differences between the two lineage differentiations, the FoxO signaling pathway was specific for OS induction and the Rap1 signaling pathway belonged only to AD induction, indicating that FoxO signaling pathway and Rap1 signaling pathway were the specific signaling pathway for the OS and AD induction, respectively. And the expression level of DEGs enriched in the FoxO signaling pathway and Rap1 signaling pathway were showed at heatmap (Fig. 3b, d).

### Key genes in the OS induction

**Fig. 4:**
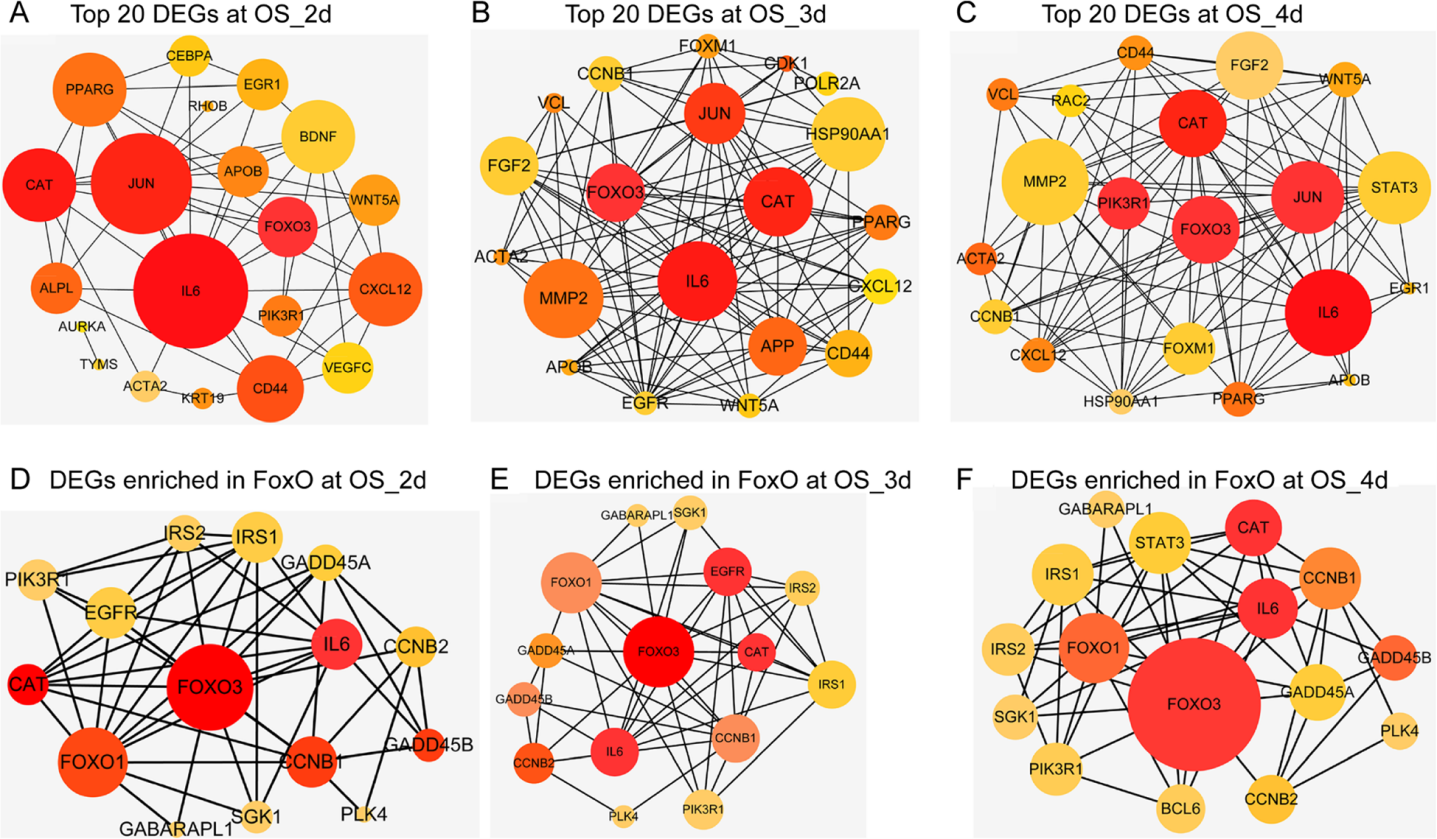
Key gene analysis in the OS. **a**–**c** Network representation of the protein-protein interactions (PPI) between the top 20 genes of the DEGs in different time points in the OS by the Between mode using cytoHubba app in the Cytoscape. The node’s colors donated the clustering coefficient, and the brighter the color, the higher the clustering, which meant stronger interaction (range from red (high) to yellow (low)), while the size of each node represented node degree distribution. **d**–**f** PPI visualization of networks with the DEGs enriched in the hub pathway (FoxO signaling pathway) of the OS, the color, and size of each point meant as the same meaning as above. **a**, **d** 2d vs 0d; **b**, **e** 3d vs 0d; **c**, **f** 4d vs 0d

Interactions between multiple genes could be well understood by PPI analysis. The DEG OS was used to perform the PPI analysis with Between and Stress algorithm of cytoHubba, from which we choose the top 20 hub genes to construct the sub-network (Fig. 4a–c and Figure S2). As shown in these six PPI networks, the common genes with high degree and clustering coefficients were FoxO3, IL-6, JUN, and CAT, which might play important roles in the OS induction. At the same time, candidate genes enriched in the FoxO signaling pathway in each group were also used to perform the PPI network (Fig. 4d–f). Similarly, FoxO1, IL-6, and CAT were localized at the center of the network. These results indicated that FoxO3, IL-6, JUN, and CAT may be the key genes for early OS differentiation. Since FoxO1, IL-6, and CAT were all enriched in the FoxO signaling pathway, it is further indicating that FoxO signaling pathway may be the specific signaling pathway for early OS differentiation.

### Key genes in the AD induction

**Fig. 5:**
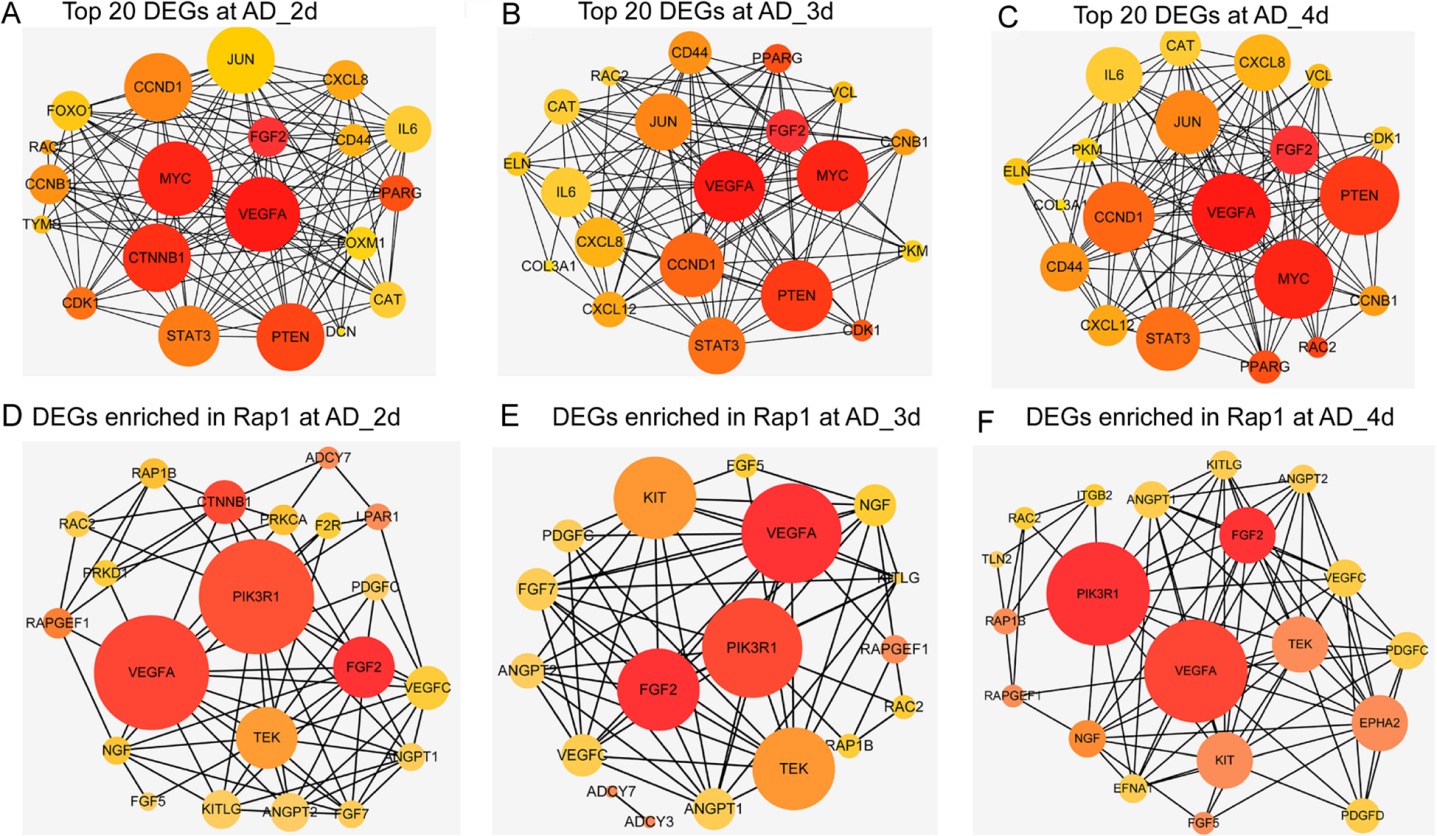
Key genes analysis in the AD. **a**–**c** Network representation of the protein-protein interactions (PPI) between the top 20 genes of the DEGs in different time points in the AD by the Between mode using cytoHubba app in the Cytoscape. The node’s colors donated the clustering coefficient, and the brighter the color, the higher the clustering, which meant stronger interaction (range from red (high) to yellow (low)), while the size of each node represented node degree distribution. **d**–**f** PPI visualization of networks with the DEGs enriched in the hub pathway (Rap1 signaling pathway) of the AD, the color, and size of each point meant as the same meaning as above. **a**, **d** 2d vs 0d; **b**, **e** 3d vs 0d; c, f 4d vs 0d

DEGs AD was used to perform the PPI networks, and sub-networks were formed by the top 20 hub genes. VEGFA, FGF2, MYC, and PTEN had the highest degree and clustering coefficients in each group computed by Between algorithm (Fig. 5a–c), while VEGFA, FGF2, MYC, CCND1, and PTEN were the core computed by Stress algorithm (Figure S3), which meant that these genes played core roles in the AD induction. Similarly, the DEGs related to Rap1 signaling pathway were selected to construct PPI network. VEGFA, FGF2, and PIK3R1 were identified as the core genes in the network (Fig. 5d–f). These interactions relied strongly on VEGFA and FGF2, which may determine the tendency of BMSCs to differentiate into AD. Therefore, hub genes VEGFA, FGF2, and Rap1 signaling pathway might be the crucial mechanisms for early AD differentiation.

### GO analysis of OS induction

**Fig. 6:**
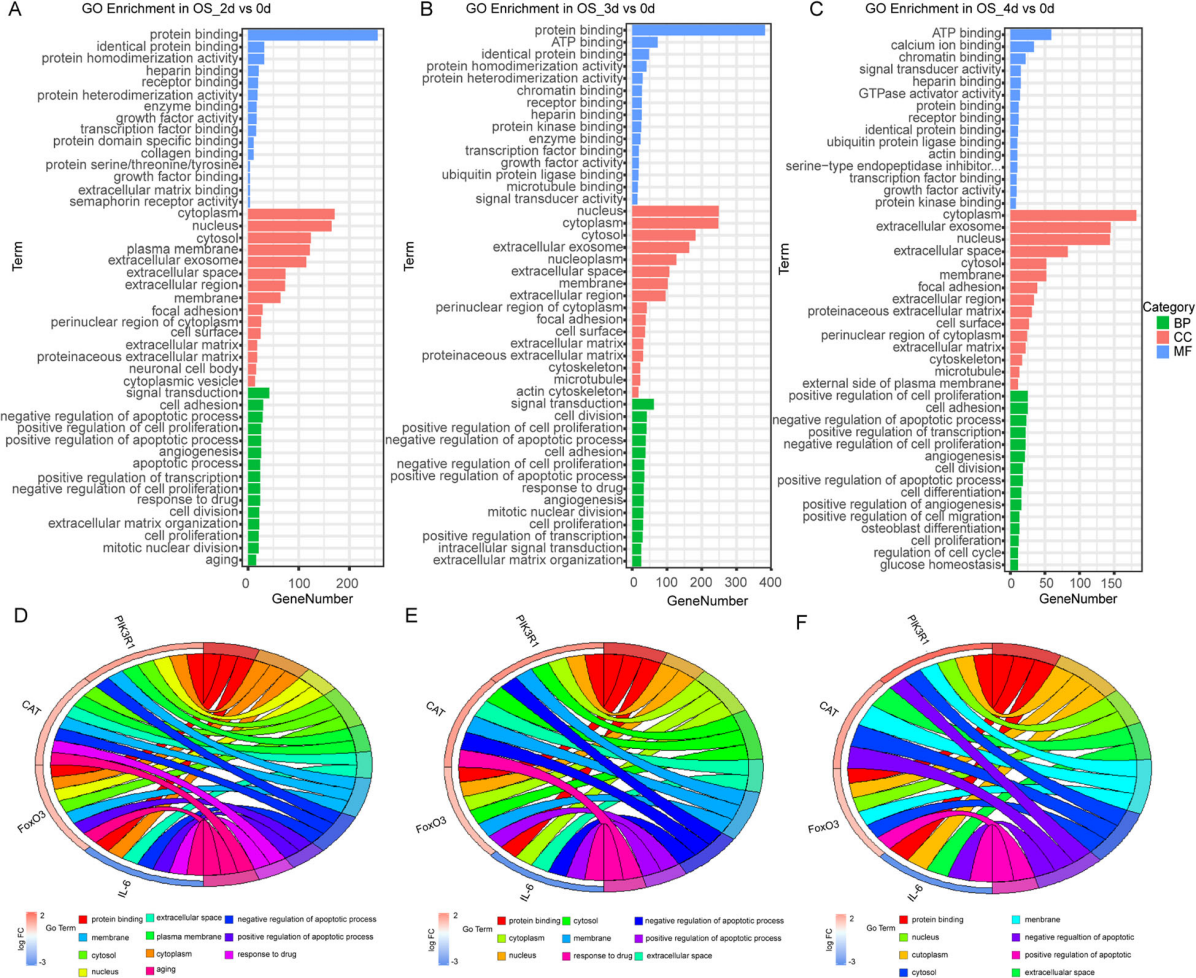
GO analysis of DEGs in the OS. **a**–**c** Top 15 of GO terms including MF, CC, and BP in the three time points. The abscissa represented the gene numbers, and the ordinate was the GO terms. (*P* value < 0.05). **d**–**f** Cord plot displays of the relationship between GO terms and key genes enriched in the hub pathway of the OS (FoxO signaling pathway). **a**, **d** 2d vs 0d; **b**, **e** 3d vs 0d; **c**, **f** 4d vs 0d; GO Gene Ontology; MF molecular function; CC cellular component; BP biological process

Gene ontology enrichment analyses were performed by the DEGs from each group, and the significant top 15 of the GO terms including BP, CC, and MF were displayed as the bar diagrams (Fig. 6a–c). In the MF category, receptor binding, protein binding, and growth factor activity were the common GO terms in the groups of OS_2d, OS_3d, and OS_4d, while cytoplasm, cytosol, and extracellular exosome were the common GO terms in the CC category. In the BP category, signal transduction, positive regulation of transcription, and positive regulation of cell proliferation were all enriched in the three time points of OS induction. To further investigate the relationship of the key genes and GO functional categories, the key genes from the FoxO signaling pathway and PI3K-Akt signaling pathway were put to perform the chord plots (Fig. 6d–f). Key genes including FoxO3, IL-6, CAT, and PIK3R1 mainly clustered into protein binding, membrane, cytosol, nucleus, extracellular space, plasma membrane, cytoplasm, negative, and positive regulation of the apoptotic process. Suggesting that GO terms in OS induction were a series of biological responses that initiated by ligand-receptor binding and transcriptional information into the nucleus.

### GO analysis of AD induction

**Fig. 7:**
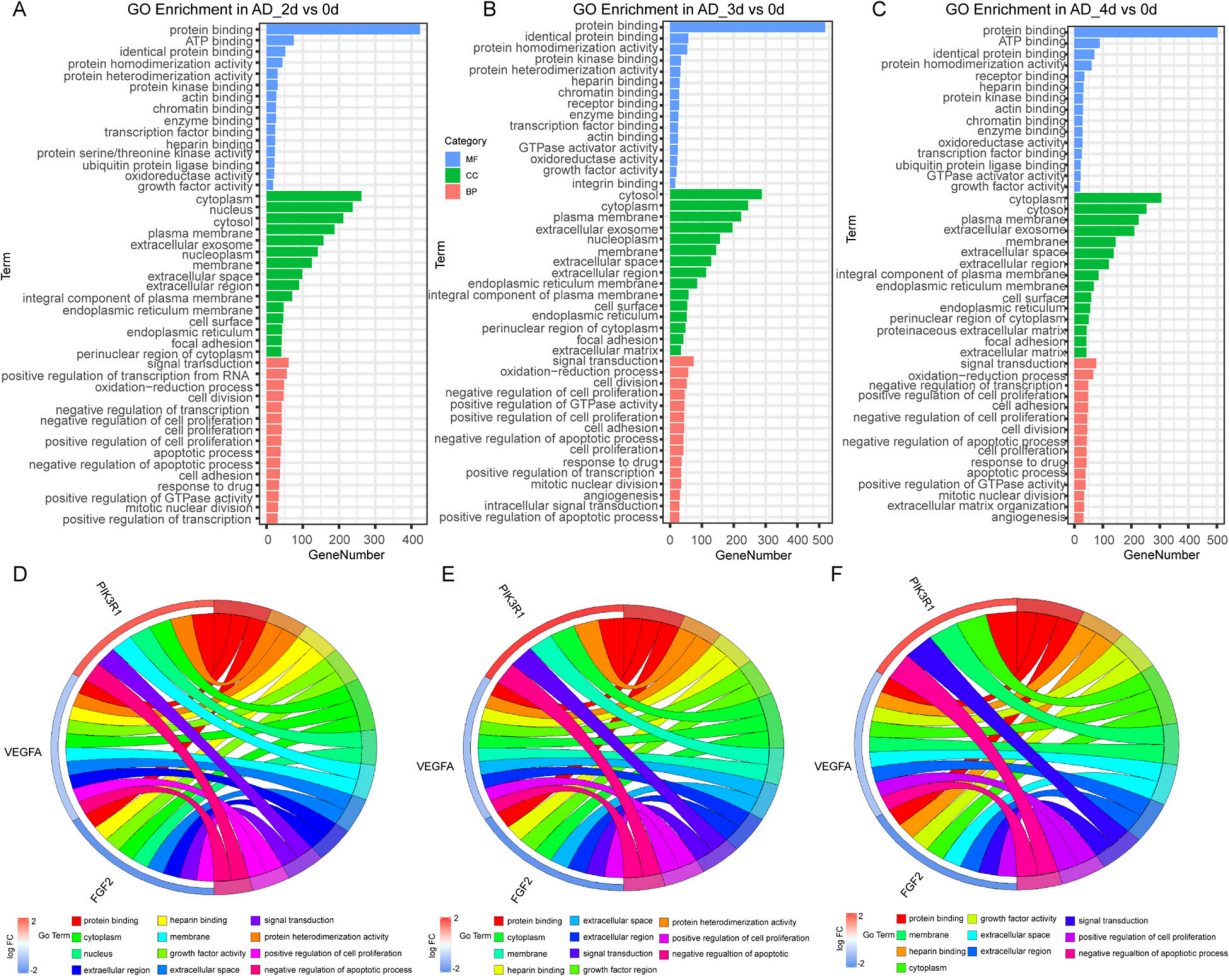
GO analysis of DEGs in the AD. **a**–**c** Top 15 of GO terms including MF, CC, and BP in the three time points. The abscissa represented the gene numbers, and the ordinate was the GO terms. (*P* value < 0.05). **d**–**f** Cord plot displays of the relationship between GO terms and key genes enriched in the hub pathway of the AD (Rap1 signaling pathway). **a**, **d** 2d vs 0d; **b**, **e** 3d vs 0d; **c**, **f** 4d vs 0d

The top 15 of the GO terms performed by the DEGs from each group of AD were shown in the bar diagrams (Fig. 7a–c). The results of the AD differentiation were similar to that in the OS, implied that there were similar biological effects on the two lineage differentiations from hMSCs. Chord plots of AD induction (Fig. 7d–f) performed by the DEGs of Rap1 signaling pathway and top 15 of GO terms in each group, showed that FGF2 was mainly enriched in the protein binding, growth factor activity, cytoplasm, nucleus, extracellular space, extracellular region, signal transduction, positive regulation of cell proliferation, and positive regulation of cell proliferation. Moreover, another target gene VEGFA was mainly related to protein binding, growth factor activity, membrane, extracellular space, extracellular region, cytoplasm, and positive regulation of cell proliferation. This was consistent with previously reported that VEGFA was an extracellular signal molecule, which regulated Rap1 signaling pathway by combining some intracellular signal factors [37, 38].

### Osteo-adipogenesis relative gene expression

**Fig. 8:**
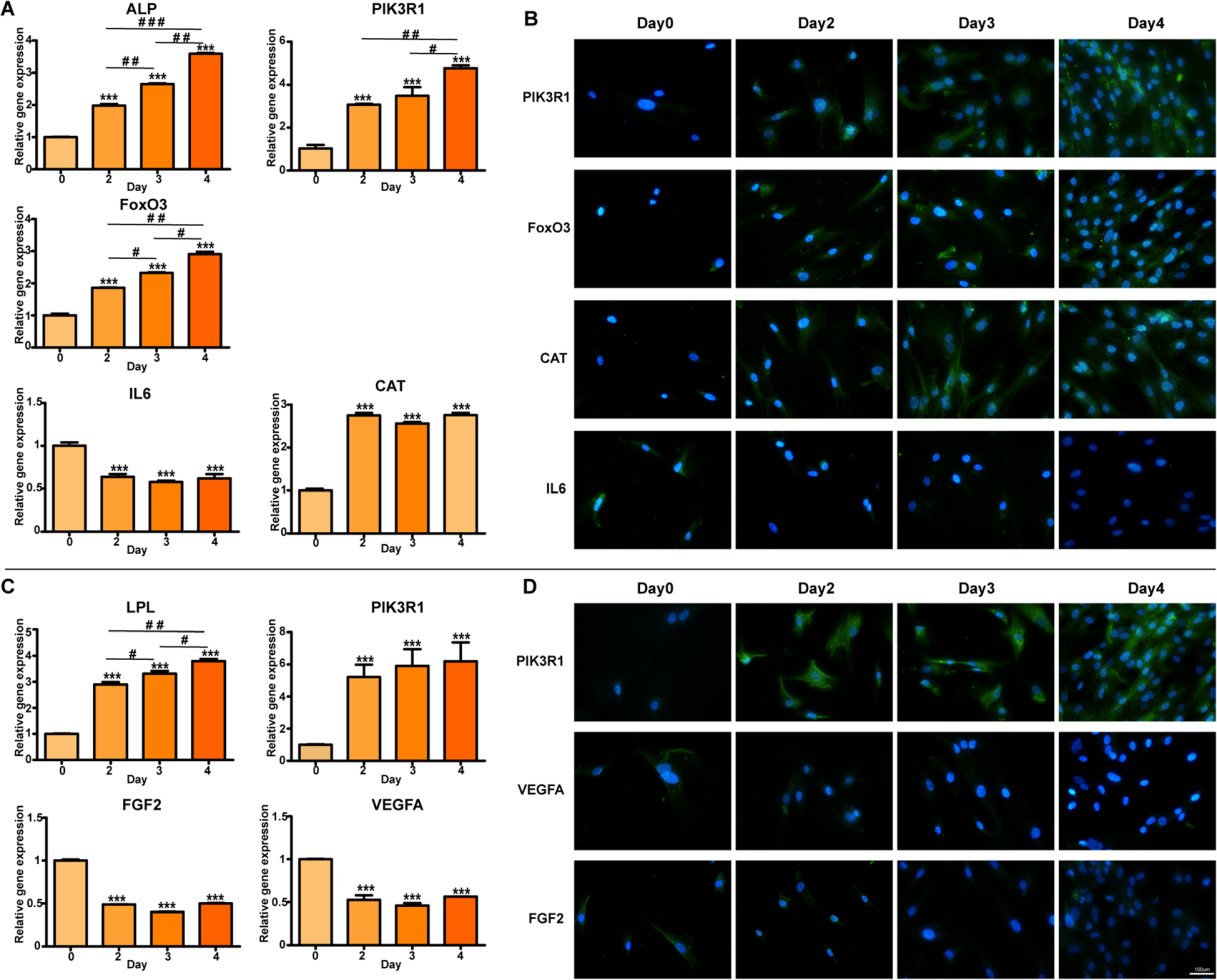
PCR and immunofluorescence staining of the key genes and proteins in the relative signaling pathways. The expression of the key genes and proteins in day 0, day 2, day 3, and day 4 (FoxO3, CAT, and IL6 in the FoxO3 signaling pathway; VEGFA and FGF2 in the Rap1 signaling pathway; PIK3R1 in PI3K-Akt signaling pathway)

The expression level of OS (ALP) and AD (LPL) specific genes at days 2–4 was increased compared to day 0, suggesting that hBMSCs were successfully induced into OS and AD (Fig. 8a, c). The expression levels of the key genes for OS (IL6 and CAT) or AD (PIK3R1, FGF2, and VEGFA) induction were changed dramatically at the first 2 days, but no obvious change was founded after that, indicating that the first 2 days may be the critical period for the lineage commitment determinant. However, FoxO3 at days 2–4 was significantly increased in the OS induction, revealing that it may be key genes for osteogenic differentiation at a steady period of lineage-continuation (Fig. 8a). In addition, the expression of PIK3R1 was higher in both OS and AD, suggesting PIK-AKT signaling pathway is a promoter in the differentiation of BMSCs.

### Signaling pathway of OS and AD induction

The expression of the key proteins in the signaling pathway relative to the OS and AD induction were detected using inflorescence staining (Fig. 8b, d). In consistent with the results of PCR, the expression of FoxO3 and CAT in OS induction at days 2–4 was higher compared to day 0, while IL6 was conversely lower, suggesting that FoxO3 signaling pathway was involved in the osteogenic differentiation (Fig. 8b). Additionally, the downregulation of VEGFA and FGF2 in AD induction indicated that Rap1 signaling pathway may be crucial for AD differentiation. Meanwhile, the expression of PIK3R1 protein was also increased both in OS and AD.

## Discussion

Loss of bone mass and aggravation of micro-fractures increase the bone fragility and susceptibility to fractures in patients of osteoporosis [39]. Currently, most strategies involving drugs based on inhibiting osteoclast activity and then blocking bone resorption were reported to cause serious adverse reactions. For example, denosumab, a new monoclonal antibody targeting RANKL, is an effective treatment of osteoporosis, but its serious side-effects including increased risk of spinal fracture and osteonecrosis, and the rebound effect after stopping denosumab exposure limit its clinical application [40–43]. Thus, there is currently a lack of enough safe and effective treatment strategy for dealing with this gap [44, 45]. Many studies have confirmed that the pathogenesis of osteoporosis is highly correlated with the tendency of increased adipocytes and decreased osteoblasts [46–48], and our analysis found the number of the DEGs in the AD was more than that of OS corresponding to previous studies (Figs. 1 and 2). Therefore, clarifying the detailed mechanisms about the differentiation of MSCs into OS and AD would be contributed to develop a new strategy and treatment for osteoporosis. As the differentiation of MSCs into a specific lineage was determined at the early stage of differentiation [49, 50], we selected early OS and AD profiles to explore the early differentiation mechanisms that determined lineage fates of BMSCs.

After a series of integration analyses, we identified FoxO3, IL-6, and CAT as key genes for osteogenic differentiation, and these genes were all enriched in the FoxO signaling pathway (Fig. 4 and S2). FoxO, an intracellular signaling factor belongs to transcriptional regulators family of forkhead box O, consisting of FoxO1, FoxO3, FoxO4, and FoxO6, plays a critical regulatory role in the multiple biological processes, including cell cycle, anti-apoptosis, and anti-oxidation [51, 52]. Previous studies had discovered that FoxO3 could reduce ROS and promote OS differentiation of MSCs by activating autophagy [53–55], which demonstrated that the upregulation of FoxO had an essential role on the activity of the OS in MSCs. Wen Sun had verified that Sirt1 overexpression promoted FoxO3a deacetylation and inhibited oxidative stress and resisted the apoptosis to increase the osteogenesis and partially restoring the defects of the skeletal system in osteoporosis [56]. However, lots of evidences also indicated that FoxO3 has a contradictory function in different tissues or expression levels. Anthony et al. had reported that the overexpression of FoxO3 could result in muscle atrophy while the ablation of FoxO3 may lead to defects in the regeneration of the muscle by downregulating MyoD (a key myogenic regulator) [57–59]. Meanwhile, Lorenowicz et al. have confirmed that activation of autophagy accelerated the osteogenic differentiation via the upregulation of FoxO3, consistent with our findings. On the contrary, stem cell lost a capacity to avoid rising ROS and osteogenic differentiation was impaired on the FoxO3 downregulation [60]. Therefore, we speculate that the function of FoxO may be dependent on the activation level and tissue location, of course, which requires further basic and animal experiments, and even future clinical experiments to discover the mechanism of the contradictory functions, revealing whether the activation of FoxO3 would bring other side effects when promoting OS in BMSCs, such as skeletal muscle atrophy. IL-6, a pleiotropic cytokine with multiple physiological functions including immune regulation, hematopoiesis, and tissue regeneration, plays a paramount role in the tissue regeneration engineer, especially in the bone metabolism [61, 62]. It could stimulate the hepatocytes to return to the cell cycle progression through triggering the initiative signal of liver regeneration [63]. Research showed that HIF1A-AS2 could erase the antagonistic effect of IL-6 exerted by miR-665 and then promote osteogenic differentiation of the BMSCs [64]. Zhongyu Xie also confirmed that IL-6 had an important role in the osteogenic differentiation of BMSCs [65]. As for CAT, Mao Li had illuminated that it could promote osteogenic differentiation through enhancing resistance to oxidative stress [66]. Next, we extracted the expression value of FOXO3, IL-6, and CAT (Fig. 8a), suggesting FOXO3 and CAT were positively correlated with osteogenic differentiation, while IL-6 was negative. Based on the previous studies, we surmised that FOXO3, IL-6, and CAT were key genes in the OS and FoxO was a core signaling pathway, especially in the early stage of OS.

In the AD, we identified hub-genes of VEGFA and FGF2 in the Rap1 signaling pathway was the essential mechanisms involved in the early stage of adipogenic differentiation (Fig. 5 and S3). Frank Yeung had indicated that Rap1, a mammalian telomere-binding protein, played a key role in the AD through its additional non-telomeric functions, which was known as co-factor of transcriptional cascade and regulator of NF-kB pathwa y[67]. VEGFA, a key angiogenic factor, was initially considered as an important molecular in the angiogenesis, and latterly was identified that it had multiple biological functions including bone cellular survival [68]. Wen Zhang pointed out that miR-128 promoted the adipogenic differentiation of hMSCs by the suppression of the VEGF pathway [69]. As a heparin-binding growth factor stored in the extracellular matrix, FGF2 had been identified to be an important modulator in the early differentiation and development of cells, owning to its multiple biological functions including chemotaxis, angiogenesis, and mitotic activity [70]. Kim et al. [71] had found that BMSCs with a deficiency of FGF2 showed a strong capacity of adipogenic differentiation, which indicated that downregulated FGF2 played an important role in the AD. Thus, VEGFA and FGF2 were both negative factors during adipogenic differentiation, which was consistent with our analysis (Fig. 8b). In the AD, FGF2 might increase the conduction of extracellular signals through molecular adhesion and then regulate the Rap1 signaling pathway to promote the differentiation of BMSCs.

In the trigger phase of BMSC differentiation, we speculated PI3K-Akt signaling pathway played a key role in triggering the differentiation of stem cells into various progenitor lines, at least in the OS and AD, based on the previous studies and our surveys (Fig. 1 and 2d–f and Fig. 3a–c). As shown in our analysis, the PI3K-Akt signaling pathway enriched the most DEGs during the whole process of the early stage in OS and AD (Figs. 1d–f and 2d–f)**,** which meant a paramount role in the differentiation of BMSCs. Activation of PI3K-Akt signaling pathway had been demonstrated not only to promote the OS and AD, but also to stimulate the chondrogenic differentiation of BMSCs, while its antagonist could lead to inhibition of BMSC differentiation [72–74]. Through the PI3K-Akt signaling pathway, BMSCs can also differentiate into other progenitor cells, such as endothelial cells (ECs) and vascular smooth muscle cells [75, 76]. Researches had confirmed that stimulation of PI3K-Akt signaling pathway had the ability to activate its downstream target of rapamycin-p70S6 kinase and promote BMSCs to differentiate into coulvascular smooth muscle. Proangiogenic bioscaffold composited of porous β-CaSiO(3)/PDLGA advanced the EC differentiation of BMSCs via activating PI3K-Akt signaling pathway, which in turn promoted phosphorylation of endothelial nitric oxide synthase (eNOS), production of nitric oxide (NO), and increased secretion of vascular endothelial growth factor (VEGF). Therefore, we surmised activation of PI3K-Akt signaling pathway in the trigger phase of BMSC differentiation might subsequently activate the FoxO and Rap1 signaling pathways, respectively, thereby promoting OS and AD. And the expression level of the key gene PIK3R1 from PI3K-Akt signaling pathway was upregulated both in the OS and AD, which are in line with our speculation (Fig. 8a, b).

Based on the GO enrichment analysis of OS, protein binding, transcription factor binding, nucleus, membrane, cytosol, and cytoplasm were clustered in the FoxO3 and IL-6. These aggregations were similar in the adipogenesis (Figs. 6d–f and 7 d–f). From these results, it seemed that osteogenic and adipogenic differentiations were consistent in biological processes, both through protein-protein binding and transmission of signaling molecules inside and outside the membrane.

## Conclusions

Taken together, this study analyzed the gene chip expression profile of hMSC differentiation to identify the potential biomarkers and key pathways between the BMSCs into OS and AD by using the bioinformatics method. Our result indicates that FoxO signaling pathway was a key pathway in the OS, and Rap1 signaling pathway is the key pathway in the AD, while the PI3K-Akt is a key signaling pathway with the key gene PIK3R1 in the initial stage of stem cell differentiation. Moreover, FOXO3, IL-6, and CAT are suggested as the potential biomarkers in the OS as well as VEGFA and FGF2 in the AD. Our research may provide a new insight into the study of BMSC differentiation, contributing to the potential therapeutic targets of osteoporosis.

## Supplementary information

**Additional file 1: Figur eS1.** Common up-and downregulations in each time points of OS and AD. (A): up of OS (B): down of OS (C): up of OS (D): down of AD. **Figure S2.** Key genesanalysis in the OS. (A-C)Network representation of the protein-protein interactions (PPI) between the top 20 genes of the DEGs in different time points in the OS by the Stress mode using cytoHubba app in the cytoscape. The node’s colors donated the clustering coefficient, and the brighter the color, the higher the clustering, which meant stronger interaction (rang from red (high) to yellow (low)), while the size of each node represented node degree distribution. (A)2d vs 0d; (B)3d vs 0d; (C)4d vs 0d. **Figure S3.** Key genes analysis in the AD. (A-C)Network representation of the protein-protein interactions (PPI) between the top 20 genes of the DEGs in different time points in the AD by the Stress mode using cytoHubba app in the cytoscape. The node’s colors donated the clustering coefficient, and the brighter the color, the higher the clustering, which meant stronger interaction (rang from red (high) to yellow (low)), while the size of each node represented node degree distribution. (A)2dvs 0d; (B)3d vs 0d; (C)4d vs 0d.

## Data Availability

The datasets generated and/or analyzed during the current study are available from the Gene Expression Omnibus repository, https://www.ncbi.nlm.nih.gov/geo/query/acc.cgi?acc=GSE80614).

## Abbreviations

BMSCs: Bone mesenchymal stem cells
AD: Adipogenesis
OS: Osteogenesis
DEGs: Differentially expressed genes
HBMSCs: Human bone mesenchymal stem cells
