# Supplementary material for: Identification of potential specific biomarkers and key signaling pathways between osteogenic and adipogenic differentiation of hBMSCs for osteoporosis therapy
Source: J Orthop Surg Res. 2020 Sep 23;15:437. doi: 10.1186/s13018-020-01965-3 (PMC7510089; doi:10.1186/s13018-020-01965-3)
Supplement: Supplementary file 1 — Additional file 1: Figur eS1. Common up-and downregulations in each time points of OS and AD. (A): up of OS (B): down of OS (C): up of OS (D): down of AD. Figure S2. Key genesanalysis in the OS. (A-C)Network representation of the protein-protein interactions (PPI) between the top 20 genes of the DEGs in different time points in the OS by the Stress mode using cytoHubba app in the cytoscape. The node’s colors donated the clustering coefficient, and the brighter the color, the higher the clustering, which meant stronger interaction (rang from red (high) to yellow (low)), while the size of each node represented node degree distribution. (A)2d vs 0d; (B)3d vs 0d; (C)4d vs 0d. Figure S3. Key genes analysis in the AD. (A-C)Network representation of the protein-protein interactions (PPI) between the top 20 genes of the DEGs in different time points in the AD by the Stress mode using cytoHubba app in the cytoscape. The node’s colors donated the clustering coefficient, and the brighter the color, the higher the clustering, which meant stronger interaction (rang from red (high) to yellow (low)), while the size of each node represented node degree distribution. (A)2dvs 0d; (B)3d vs 0d; (C)4d vs 0d. [file 13018_2020_1965_MOESM1_ESM.pdf]

Supplementary Material

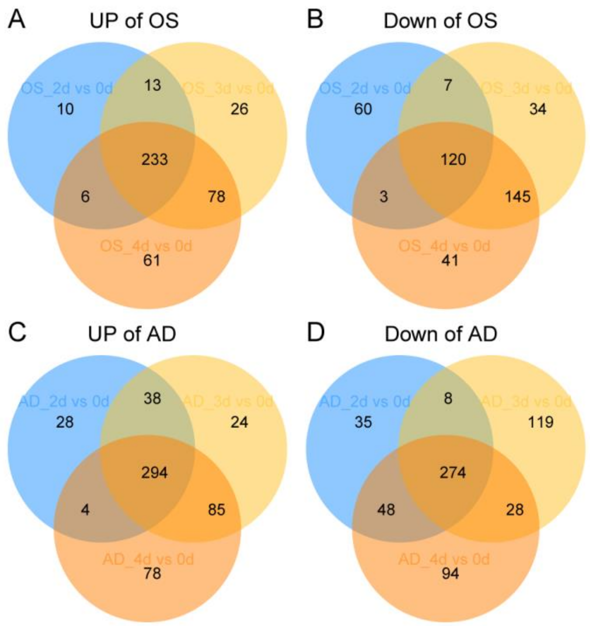

Figure S1. Common up-and downregulations in each time points of OS and AD. (A): up of OS (B): down of OS (C): up of OS (D): down of AD.

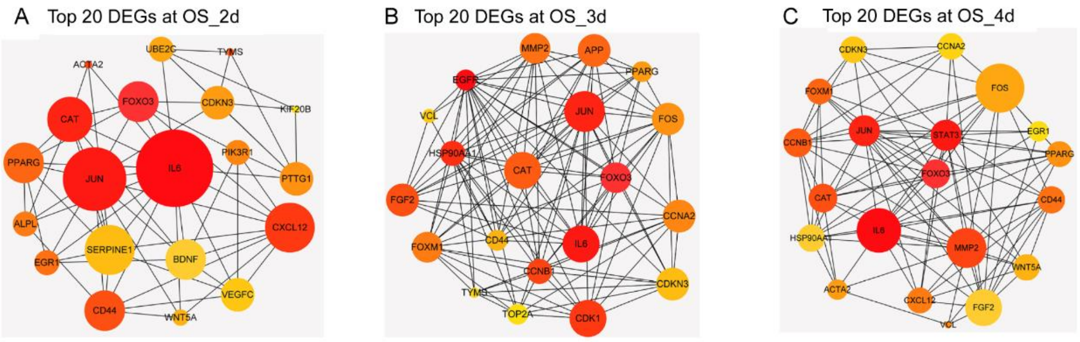

Figure ~~S1~~S2. Key genes analysis in the OS. (A-C) Network representation of the protein-protein interactions (PPI) between the top 20 genes of the DEGs in different time points in the OS by the Stress mode using cytoHubba app in the cytoscape. The node's colors donated the clustering coefficient, and the brighter the color, the higher the clustering, which meant stronger interaction (rang from red (high) to yellow (low)), while the size of each node represented node degree distribution. (A) 2d vs 0d; (B) 3d vs 0d; (C) 4d vs 0d.

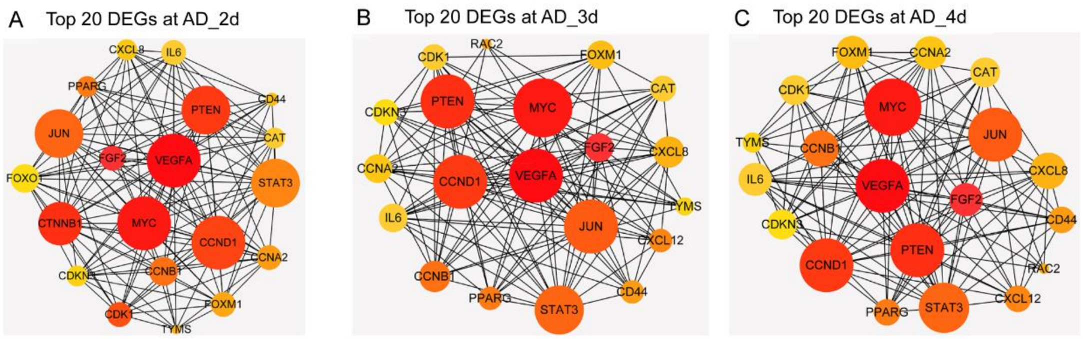

Figure ~~S2~~S3. Key genes analysis in the AD. (A-C) Network representation of the protein-protein interactions (PPI) between the top 20 genes of the DEGs in different time points in the AD by the Stress mode using cytoHubba app in the cytoscape. The node's colors donated the clustering coefficient, and the brighter the color, the higher the clustering, which meant stronger interaction (rang from red (high) to yellow (low)), while the size of each node represented node degree distribution. (A) 2d vs 0d; (B) 3d vs 0d; (C) 4d vs 0d.
